# Predictive Biomarkers for Response and Toxicity of Induction Chemotherapy in Head and Neck Cancers

**DOI:** 10.3389/fonc.2022.900903

**Published:** 2022-07-06

**Authors:** Łukasz Boguszewicz

**Affiliations:** Department of Medical Physics, Maria Sklodowska-Curie National Research Institute of Oncology, Gliwice Branch, Warszawa, Poland

**Keywords:** induction chemotherapy, HNSCC (head and neck squamous cell carcinoma), biomarkers, prediction, outcome, toxicity

## Abstract

This review focuses on the molecular biology of head and neck squamous cell carcinomas and presents current and emerging biomarkers of the response of patients to induction chemotherapy. The usefulness of genes, proteins, and parameters from diagnostic clinical imaging as well as other clinicopathological parameters is thoroughly discussed. The role of induction chemotherapy before radiotherapy or before chemo-radiotherapy is still debated, as the data on its efficacy are somehow confusing. Despite the constant improvement of treatment protocols and the introduction of new cytostatics, there is still no consensus regarding the use of induction chemotherapy in the treatment of head and neck cancer, with the possible exception of larynx preservation. Such difficulties indicate that potential future treatment strategies should be personalized. Personalized medicine, in which individual tumor genetics drive the selection of targeted therapies and treatment plans for each patient, has recently emerged as the next generation of cancer therapy. Early prediction of treatment outcome or its toxicity may be highly beneficial for those who are at risk of the development of severe toxicities or treatment failure—a different treatment strategy may be applied to these patients, sparing them unnecessary pain. The literature search was carried out in the PubMed and ScienceDirect databases as well as in the selected conference proceedings repositories. Of the 265 articles and abstracts found, only 30 met the following inclusion criteria: human studies, analyzing prediction of induction chemotherapy outcome or toxicity based on the pretreatment (or after the first cycle, if more cycles of induction were administered) data, published after the year 2015. The studies regarding metastatic and recurrent cancers as well as the prognosis of overall survival or the outcome of consecutive treatment were not taken into consideration. As revealed from the systematic inspection of the papers, there are over 100 independent parameters analyzed for their suitability as prognostic markers in HNSCC patients undergoing induction chemotherapy. Some of them are promising, but usually they lack important features such as high specificity and sensitivity, low cost, high positive predictive value, clinical relevance, short turnaround time, etc. Subsequent studies are necessary to confirm the usability of the biomarkers for personal medicine.

## 1 Introduction

Head and neck squamous cell carcinomas (HNSCCs) develop in organs that play pivotal roles in respiratory, nutritional, and social functions. Thus, a crucial goal of HNSCC treatment is organ preservation. One of the most important cancer treatment methods is systemic chemotherapy (so-called induction chemotherapy, iCHT) (1, 2). In the advanced stages of the disease (locally advanced head and neck squamous cell carcinoma, LA-HNSCC), it makes a subsequent treatment (such as surgery, radiotherapy, or chemoradiotherapy) more effective and significantly supports organ preservation. LA-HNSCC patients with a high risk of distant failure, multiple involved nodes, or large-volume nodal disease appear to gain certain benefits from this chemotherapy approach (3).

However, iCHT, as any systemic treatment, is burdened with relatively high toxicity (4–6) and there is still room for improvement in its effectiveness. Complete response (CR) to iCHT (disappearance of target lesions) is observed in around 30% of patients. Partial response (PR, a reduction of at least 30% in the sum of the longest diameter of target lesions) usually reaches up to or is slightly more than 60%, while the rest of the patients treated with iCHT present progressive (PD, an increase of at least 20% in the sum of the longest diameter of the target lesions) or stable disease (SD, unqualified to PR or PD) (7–10).

It is still unclear where the individual differences in response to chemotherapy come from, but the effectiveness of this treatment largely depends on giving the patient the full number of cycles, which is often impossible due to high toxicity. Thus, finding predictive markers of toxicity and pathological response to iCHT is of great importance. In 2015, Cosway et al. (11) published an article entitled “Biomarkers predicting chemotherapy response in head and squamous cell carcinoma: a review.” They concluded that: “The role of biomarkers in the induction and neoadjuvant setting is not yet well established. Several biomarkers have been proposed, but no markers are currently in clinical use. Future research should involve collaboration with basic science colleagues for developing novel biomarkers for head and neck cancer.”

The purpose of this review is to evaluate the progress made in the field over the past six years.

## 2 Methods

This study was designed in accordance with the Preferred Reporting Items for Systematic Reviews and Meta-Analyses (PRISMA) statement (http://www.prisma-statement.org/). An extensive search for research articles published since 2015 was performed in two search engines: PubMed and ScienceDirect. An additional search included the proceedings of the meetings of the European Society for Medical Oncology (ESMO), European Society for Therapeutic Radiation and Oncology (ESTRO), American Society of Clinical Oncology (ASCO), and American Society for Radiation Oncology (ASTRO) as the most relevant to the subject.

### 2.1 Search Strategy and Data Extraction

The data search and extraction strategies were based on the criteria that defined the boundaries of this systematic review. The search criteria were developed to narrow the search to academic papers or conference abstracts. The queries included multiple logical operators, nested clauses, and the term “*” with a wildcard character to cover various papers on the topics identified by the inclusion criteria.

The following criteria had to be met for inclusion:

- Peer-reviewed research article or conference abstract. The year of publication was from 2015 to the present.
- Human study.
- Treatment of HNSCC with iCHT.
- Prediction of iCHT outcome or toxicity based on the pretreatment (or after the first iCHT cycle, if more cycles were administered) data.

A PubMed search was carried out in Advanced Search Builder. Two search queries were formulated according to the inclusion criteria and applied:

- (“induction chemotherapy”[Title] OR “induction”[Title] OR “neoadjuvant”[Title] OR “TPF”[Title] OR “chemotherapy”[Title] OR “cisplatin”[Title] OR “PF”[Title] OR “paclitaxel”[Title] OR “5-fluorouracil”[Title] OR “carboplatin”[Title]) AND ((“biomark*”[Title]) OR (“marker*”[Title]) OR (“predict*”[Title]) OR (“predictor*”[Title]) OR (“prognos*”[Title])) AND ((“squamous cell carcinoma of head and neck”[MeSH Terms] OR (“squamous”[All Fields] AND “cell”[All Fields] AND “carcinoma”[All Fields] AND “head”[All Fields] AND “neck”[All Fields]) OR “squamous cell carcinoma of head and neck”[All Fields] OR (“head”[All Fields] AND “neck”[All Fields] AND “squamous”[All Fields] AND “cell”[All Fields] AND “carcinomas”[All Fields]) OR “head and neck squamous cell carcinomas”[All Fields])) NOT (meta-analysis[pt] OR review[pt] OR meta-analysis[ti] OR systematic literature review[ti] OR systematic review[ti] OR literature review[ti]) NOT (“recurrent”[Title] OR “metastatic”[Title] OR “salvage”[Title]).
- (“induction chemotherapy”[tw] OR “induction chemotherapy”[Mesh]) AND (“metabolomics”[tw] OR “metabonomics”[tw] OR “metabolomics”[Mesh]) NOT (meta-analysis[pt] OR review[pt] OR meta-analysis[ti] OR systematic literature review[ti] OR systematic review[ti] OR literature review[ti]) NOT (“recurrent”[Title] OR “metastatic”[Title] OR “salvage”[Title]).

ScienceDirect database was searched using the advanced search form. The search was carried out in the title, abstract and keywords, and in the absence of results, also in the entire manuscript.

The following queries were used:

- (“induction chemotherapy” OR “neoadjuvant chemotherapy”) AND (biomarker OR marker OR predictor OR prediction OR prognostic) AND (head and neck OR HNSCC)
- (“TPF” OR “PF”) AND (biomarker OR marker OR predictor OR prediction OR prognostic) AND (head and neck OR HNSCC)
- (“induction chemotherapy” OR “neoadjuvant chemotherapy”) AND (head and neck OR HNSCC) AND (“metabolomics”).

The conference abstracts were searched in the databases of Annals of Oncology (ESMO), Radiotherapy & Oncology (ESTRO), International Journal of Radiation Oncology, Biology, Physics (ASTRO), and ASCO Meeting Library using the same search queries as in the case of the ScienceDirect database.

The results were manually checked for relevance to the research question; duplicates and those not meeting the inclusion criteria were excluded. The titles and abstracts of all records were appraised to identify eligible studies. When the abstract was not sufficient to determine the relevance, the full texts of the selected papers were assessed.

At this stage, the exclusion criteria were as follows:

- Metastatic or recurrent HNSCC.
- Other types of cancer or different diseases.
- Non-human studies.
- Patients not receiving iCHT.
- No prognosis of iCHT outcome (assessed before the consecutive treatment) or toxicity.
- Review articles, commentaries, etc.

## 3 Results

**Figure 1:**
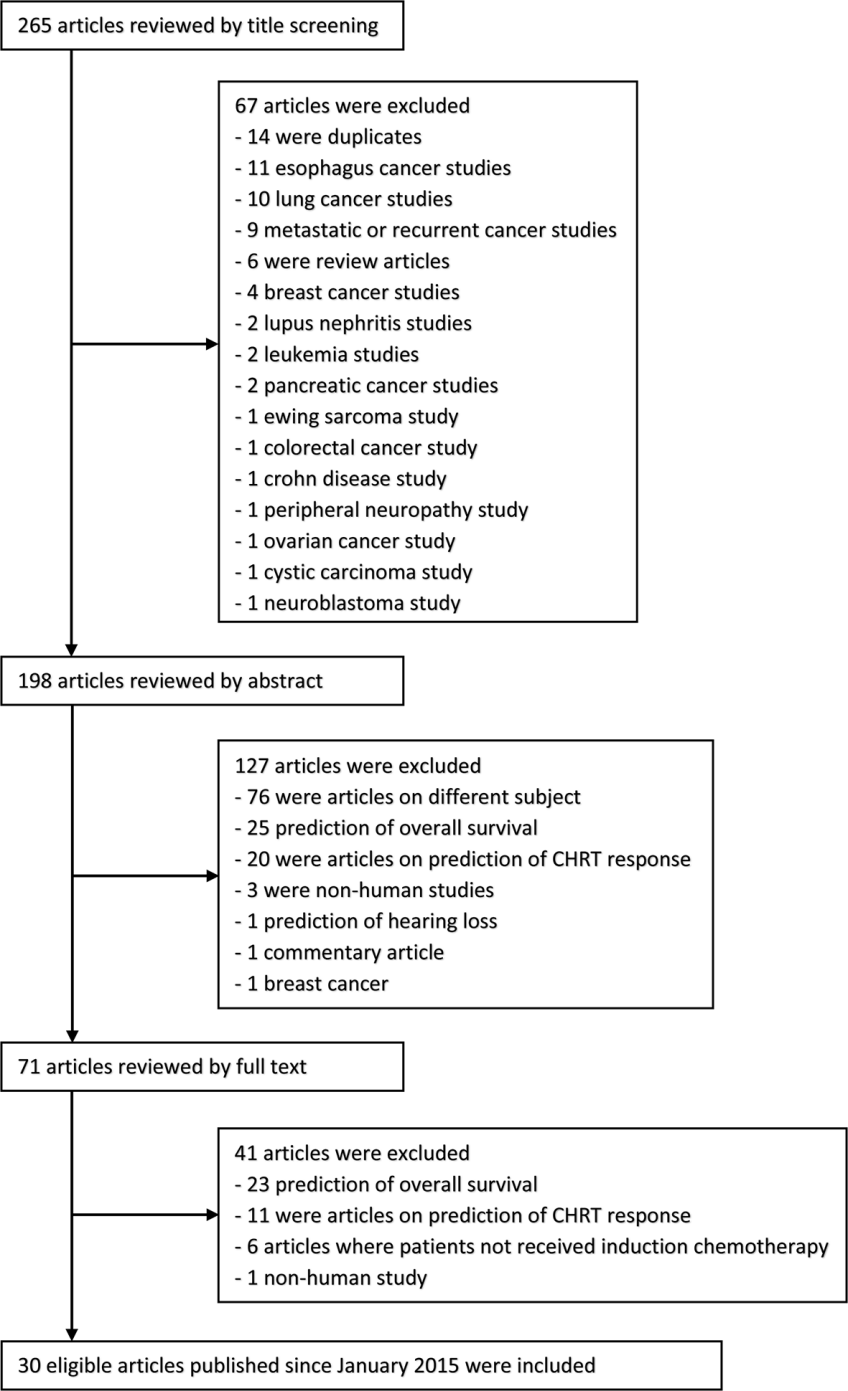
The flow diagram of the selection process.

The first search of the PubMed database resulted in 80, the latter one in 7 positions. The three search queries in ScienceDirect resulted in 32, 9, and 17 positions, respectively. A total of 120 abstracts were found in the conference databases. After screening the titles, abstracts and the full texts of 265 publications and conference abstracts identified, 235 were excluded because of duplication, obvious irrelevance, and the inconsistency in the inclusion criteria. Finally, 30 papers (and none of the conference abstracts) were analyzed. The flowchart of the process of searching for works related to the defined criteria is illustrated in **Figure 1**.

The eligible studies have been grouped and discussed by the methodology used. The results related to the search for prognostic factors of response to treatment and for toxicity of iCHT are analyzed in the separate sections.

### 3.1 Identification of Prognostic Factors for Response to iCHT Treatment

The response to iCHT is usually measured as the degree of the primary tumor and/or node shrinkage. Although the scales used by different authors to assess the effectiveness of treatment may vary, the most commonly used is the so-called RECIST (Response Evaluation Criteria in Solid Tumors) scale. Currently, RECIST 1.1 is the gold standard for assessing treatment response in solid tumors (12). Key features of RECIST include the definitions of the minimum size for a measurable lesion, the instructions on how many lesions to follow, and the use of unidimensional, rather than bidimensional, measures for the overall evaluation of tumor burden. If the authors explicitly referred to the RECIST guidelines for assessing the iCHT response, an appropriate annotation is included in **Tables 1** **–** **4** summarizing the results of genetic, protein-based, diagnostic imaging, and clinicopathological studies.

**Table 1:** Summary of genetic studies on predicting iCHT response.

Ref.	Gene	Gene name	RS	N-RS	Tumor localization	No. of patients	Assessment of iCHT response
(13)	GATS	stromal antigen 3 opposite strand	↑		Hypopharynx(n = 29)	29	Responders* (n = 16): Tumor volume decreasedapprox. 70%.**Non-responders (n = 13): **Tumor volume decreased less than approx. 25%.Tumor volume decreasedbetween 25 and 75% was excluded from the study.
	PRIC285	helicase with zinc finger 2		↑			
	ARID3B	AT-rich interaction domain 3B	↑				
	ASNS	asparagine synthetase (glutaminehydrolyzing)	↑				
	CXCR1	C-X-C motif chemokine receptor 1		↑			
	FBN2	fibrillin	↑				
	INMT	indolethylamine N-methyltransferase		↑			
	MYOM3	myomesin 3		↑			
	SLC27A5	solute carrier family 27 (fatty acid transporter), member 5	↑				
	STC2	stanniocalcin 2	↑				
(14)	TS	thymidylate synthase			Oropharynx(n = 30)Hypopharynx(n = 34)	64	Responders* (n = 21): CR.**Non-responders (n = 43):** other.
	DPD	dihydropyrimidine dehydrogenase	↑				
	OPRT	orotate phosphoribosyltransferase					
	TP	tymidine phosphorylase					
	MDR1	multidrug resistance gene 1					
	MRP1	multidrug resistance−associated protein 1	↑				
	COX2	cyclooxygenase−2					
	EGFR	epidermal growth factor receptor					
	HER2	human epidermal growth factor receptor 2					
	VEGF	vascular endothelial growth factor	↑				
	Bcl-2	b−cell lymphoma 2	↑				
	Rb1	RB Transcriptional Corepressor 1					
	E2F1	E2F Transcription Factor 1					
	GST−pi	glutathione S−transferase−pi					
	ERCC1	excision repair cross−complementing 1	↑				
	XPA	xeroderma pigmentosum	↑				
	ENT1	equilibrative nucleoside transporter 1					
	β-tubulin	β-tubulin					
	p53	P53	↑				
	Bcl-xL	b−cell lymphoma−extra large					
	PIK3CA	phosphoinositide 3−kinase					
	PTEN	phosphatase and tensin homolog					
(15)	MAPK10	mitogen-activated protein kinase 10	↑		Larynx(n = 57)	57	Responders* (n = 21): CR + PR.**Non-responders (n = 36): **SD + PD.
	c-Jun	c-Jun	↑				
	Itga6	integrin alpha-6		↑			
(16)	SHH	sonic hedgehog		↑	No data ^1^	53	**Responders: **CR.**Non-responders: **other.
	Nrf2	nuclear factor erythroid 2-related factor 2		↑			

CR, complete response; PR, partial response; SD, stable disease; PD, progressive disease. RS - responders, N-RS - non-responders.

^1^Clinical characteristic was given for the entire study group, while patients treated with induction chemotherapy constituted a small subset.

*Response to iCHT assessed using RECIST guidelines. ­ ↑higher value predicts better (when in RS column) or worse (when in N-RS column) response.

**Table 2:** Summary of protein-based studies on predicting iCHT response.

Ref.	Protein	Protein name	RS	N-RS	Tumor localization	No. of patients	Assessment of iCHT response
(29)	TCN1	Transcobalamin I		↑	Hypopharynx (n = 102)	102	**Responders (n = 75):** CR + PR.**Non-responders (n = 27):** SD + PD.
(30)	p16	p16			Hypopharynx (n = 45)	45	**Responders(n = 17):** CR or **(n = 41):** CR + PR.**Non-responders(n = 28):** Non-CR or **(n = 4):** Non-CR + PR.
	p53	p53					
(31)	p16	p16			Oropharynx (n = 33)Hypopharynx (n = 37)Larynx (n = 11)	81	Responders* (n = 50):≥80% decrease of initial tumor size.**Non-responders (n = 31):**<80% decrease of initial tumor size.
	p53	p53					
(32)	p16	p16			Oropharynx (n = 19)Hypopharynx (n = 15)Larynx (n = 6)Oral cavity (n = 10)	47^1^	**Responders (n = 33):** PR.**Non-responders (n = 14):** SD + PD.
(33)	PITX1	pituitary homeobox 1	↑^2^		Larynx (n = 21), Hypopharynx (n = 16)Oropharynx (n = 5), Oral (n = 5)	47	CR* (n = 6), PR (n = 21), SD + PD (n = 20)
	p53	p53					
(34)	Notch1	single-pass transmembrane protein encoded by the NOTCH gene		↑	Larynx (n = 41)Hypopharynx (n = 13)Oropharynx (n = 8)Tongue, gingiva, nasal sinuses (n = 8)	72	Responders* (n = 40): CR + PR.**Non-responders (n = 32):** SD + PD.
(35)	LOXL4	lysyl oxidase-like 4			No data^3^	25	**Responders (n = 21):**≥50% reduction in tumor diameter.**Non-responders (n = 4):**<50% reduction in tumor diameter.
(15)	MAPK10	mitogen-activated protein kinase 10	↑		Larynx (n = 57)	57	Responders* (n = 21): CR + PR.** Non-responders (n = 36):** SD + PD.
	c-Jun	c-Jun	↑				
	Itga6	integrin alpha-6	↑				

CR, complete response; PR, partial response; SD, stable disease; PD, progressive disease. *Response to iCHT assessed using RECIST guidelines.

^1^Response to induction chemotherapy was available in 47 of 50 patients. RS - responders, N-RS - non-responders.

^2^PITX1 was higher in CR compared to SD + PD, no differences in PR were observed.

^3^Clinical characteristic was given for the entire study group, while patients treated with induction chemotherapy constituted a small subset. ↑ higher value predicts better (when in RS column) or worse (when in N-RS column) response.

**Table 3:** Summary of diagnostic imaging studies on predicting iCHT response.

Ref.	Parameter	Definition	RS	N-RS	Tumor localization	No. of patients	Assessment of iCHT response
(41)	SUVmax reduction after one iCHT cycle	Reduction of maximum standardized uptake value (FDG PET)	↑		Piriform sinus (n = 15)Oropharynx (n = 6)	21	**Responders (n = 13):**tumor volume decreased ≥70%.**Non-responders (n = 8):**tumor volume decreased <70%.
(42)	SUVmax	Maximum standardized uptake value (FDG PET)			OropharynxHypopharynxOral cavity	35	**Responders (n = 22):**CR + PR.**Non-responders (n = 13):**SD + PD.
	MTV	Metabolic tumor volume (FDG PET)					
(43)	SUVmax	Maximum standardized uptake value (FDG PET)			Larynx	62	Responders* (n = 48):tumor surface shrinkage ≥30%.**Non-responders (n = 14):**tumor surface shrinkage <30%.
	preCT	maximum axial tumor diameter (CT)					
(44)	SUVmax	Maximum standardized uptake value (FDG PET)			LarynxHypopharynx	49	**Responders (n = 39):**tumor surface shrinkage ≥30%.**Non-responders (n = 10):**tumor surface shrinkage <30%.
	SUVmean	Mean standardized uptake value (FDG PET)					
	MTV	Metabolic tumor volume (FDG PET)					
	VT	Volume of the primary tumor (CT)					
	VN	Volume of the lymph nodes (CT)					
	V	VT + VN (CT)					
(45)	Tumor Fp	Plasma flow in tumor (DCE-MRI)	↑		Tonsil(n = 15)Base of tongue(n = 11)Hypopharynx(n = 6)Oral tongue(n = 2)Naspoharynx(n = 2)Glottic larynx(n = 1)	37	Responders* (n = 25):PR.**Non-responders (n = 12):**SD.
	Tumor PS	Permeability-surface area product in tumor (DCE-MRI)					
	Tumor vp	Plasma volume in tumor (DCE-MRI)					
	Tumor ve	Volume of extravascular extracellular space in tumor (DCE-MRI)					
	Tumor K^trans^	K^trans^ in tumor (DCE-MRI)					
	Nodes Fp	Plasma flow in lymph nodes (DCE-MRI)					
	Nodes PS	Permeability-surface area product in lymph nodes (DCE-MRI)					
	Nodes vp	Plasma volume in lymph nodes (DCE-MRI)					
	Nodes ve	Volume of extravascular extracellular space in lymph nodes (DCE-MRI)					
	Nodes K^trans^	K^trans^ in lymph nodes (DCE-MRI)					
(46)	Mean ADC	Mean apparent diffusion coefficientin tumor (DW- MRI)		↑	Oral cavity(n = 8)Oropharynx(n = 10)Nasopharynx(n = 5)Larynx(n = 1)Maxillary sinus(n = 1)	25	Responders* (n = 13):CR + PR.**Non-responders (n = 12):**SD + PD.
	Kurtosis	The degree of peakedness of ADC distribution in tumor (DW-MRI)					
	Skweness	A measure of the degree of assymetry of ADC distribution in tumor (DW-MRI)					
(47)	MPI	Minimum pixel intensity in lymph nodes (CT)			No data	27 (41^1^)	**Responders (n = 20):**Reduction in lymph node volume >66%.**Non-responders (n = 21):**Reduction in lymph node volume ≤66%.
	Skewness	A measure of the degree of assymetry of intensity distribution in lymph nodes (CT)					
	LGRE	Low Gray Level Run Emphasis in lymph nodes (CT)					
(48)	SLU	Score of liver ultrasonography			Oropharynx(n = 16)Hypopharynx(n = 16)Larynx(n = 3)Oral cavity(n = 12)	47	Responders* (n = 25):CR + PR.**Non-responders (n = 13):**SD + PD.9 patients died before the evaluation.

CR, complete response; PR, partial response; SD, stable disease; PD, progressive disease. RS - responders, N-RS - non-responders.

^1^41 enlarged lymph nodes from 27 patients were studied.

*Response to iCHT assessed using RECIST guidelines. ­ ↑ higher value predicts better (when in RS column) or worse (when in N-RS column) response.

**Table 4:** Summary of studies analyzing clinicopathological parameters in regard of predicting iCHT response.

Ref.	Parameter	Definition	RS	N-RS	Tumor localization	No. of patients	Assessment of iCHT response
(31)	Localization	Primary tumor localization	↑^1^		Oropharynx (n = 33)Hypopharynx (n = 37)Larynx (n = 11)	81	Responders* (n = 50):≥80% decrease of initial tumor size.**Non-responders (n = 31):**<80% decrease of initial tumor size.
	SR	Stromal reaction					
	HTT	Histologic type of tumor					
	LHR	Lymphocytic host response	↑				
	NC	Neutrophil count					
	LC	Lymphocyte count					
	NLR	Neutrophil to lymphocyte ratio					
	PLR	Platelet to lymphocyte ratio		↑			
	WPOI	Worst pattern of invasion					
	B–G risk	Brandwein-Gensler risk		↑			
(32)	CD8	CD8 T lymphocytes			Oropharynx (n = 19)Hypopharynx (n = 15)Larynx (n = 6)Oral cavity (n = 10)	35-40^2^	**Responders (n = 33):**PR.**Non-responders (n = 14):**SD + PD.
	CD16	Fcγ receptor III					
	CD20	CD20 B cells					
	CD68	CD68 macrophages					
	CD134	CD134 T lymphocytes					
	CD137	CD137 T lymphocytes					
	DC-LAMP	DC-LAMP mature dendritic cells	↑				
	FOXP3	FOXP3 lymphocytes					
	MPO	myeloperoxidase+ neutrophil granulocytes					
	NKp46	NKp46 natural killer cells					
	PD-1	PD-1 lymphocytes	↑				
	NC	Neutrophil count	↑				
	LC	Lymphocyte count					
(52)	NLR	Neutrophil to lymphocyte ratio			Larynx (n = 36)Hypopharynx (n = 28)	64	Responders* (n = 26):>50% decrease in the larger dimension of the primary tumor.**Non-responders (n = 38):**≤50% decrease in the larger dimension of the primary tumor.
	CD8	CD8 T lymphocytes					
	FOXP3	FOXP3 lymphocytes					
	CD8/FOXp3		↑				
	PD-L1 TPS	Programmed cell death ligand 1 tumor proportion score					
	PD-L1 CPS	Programmed cell death ligand 1 combined proportion score		↑			
	NLR/PD-L1 CPS			↑			
(53)	NC	Neutrophil count			Hypopharynx (n = 72)	72	Responders* (n = 52):CR + PR.**Non-responders (n = 20):**SD + PD.
	PC	Platelet count					
	LC	Lymphocyte count		↑			
	MC	Monocyte count					
	PLR	Platelet to lymphocyte ratio	↑				
	NLR	Neutrophil to lymphocyte ratio	↑				
(54)	CD4	CD4 T lymphocytes	↑		Hypopharynx (n = 40)	40	Responders* (n = 26):CR + PR.**Non-responders (n = 14):**SD + PD.
	CD8	CD8 T lymphocytes	↑				
	Tregs	Regulatory T cells		↑			
	NK cells	Natural killer cells					
(55)	CTCs	Tumor circulating cells			Oropharynx (n = 40)	40	Responders* (n = 8):CR.**Non-responders (n = 32):**Non-CR.
(56)	BMI	Body mass index				109	**No data**
(13)	TNM	Clinical stage			Hypopharynx (n = 29)	29	Responders* (n = 16):tumor volume decreased approx. 70%.**Non-responders (n = 13):**tumor volume decreased less than approx. 25%.Tumor volume decreased between 25 and 75% was excluded from the study.
(14)	T	Primary tumor stage		↑	Oropharynx (n = 30)Hypopharynx (n = 34)	64	Responders* (n = 21:CR.**Non-responders (n = 43):**other.
	N	Nodal stage					
(46)	T	Primary tumor stage			Oral cavity (n = 8)Oropharynx (n = 10)Nasopharynx (n = 5)Larynx, Maxillary sinus (n = 2)	25	Responders* (n = 13):CR + PR.**Non-responders (n = 12):**SD + PD.
	TV	Tumor volume in cm3		↑			
(57)	T	Primary tumor stage			Hypopharynx (n = 19)Oropharynx (n = 60)Oral cavity (n = 62)Larynx (n = 17),Unknown (n = 4)	162	Responders* (n = 99):CR + PR.**Non-responders (n = 63):**SD + PD.
	N	Nodal stage		↑			

CR, complete response; PR, partial response; SD, stable disease; PD, progressive disease. RS - responders, N-RS - non-responders.

^1^Responders were mostly patients with hypopharynx cancer.

^2^Number of biopsy samples with sufficient amount of tumor tissue was lower than the number of patients.

*Response to iCHT assessed using RECIST guidelines. ↑ higher value predicts better (when in RS column) or worse (when in N-RS column) response.

#### 3.1.1 Genes

Tumors are complex ecosystems that evolve in response to intrinsic and extrinsic perturbations, and gene expression is regulated by both. The extrinsic mechanisms originate from unequal microenvironments, whereas the intrinsic mechanisms include cell-to-cell variability in genotypic alterations and non-genetic or phenotypic variations, which are due to epigenetic modification, plastic gene expression, and signal transduction (17). Recent studies confirm that epithelial tumors, such as HNSCC, can be generated through cell-extrinsic means *via* the microenvironment (18–20) and that both cell-intrinsic and cell-extrinsic factors generate intra-tumor heterogeneity (21).

Tumor genotype variations among different patients are known as interpatient heterogeneity (IPH), whereas the genetic heterogeneity within a tumor is called intra-tumor heterogeneity (ITH). ITH denotes a substantial variation at the genetic, epigenetic, and phenotypic levels (22). The impact of such diversification on metastatic potential is still unknown. However, there is a growing recognition that heterogeneity and evolution play a significant role in driving treatment failure (23). Clonal mutations are shared by all cancer cells, whereas sub-clonal ones are present only in a subset (24)—tumor progression depends on the balance between the phenotypically and spatially well-defined hierarchies of the tumor clones (25) and can even select for more aggressive clones in advanced and more anaplastic stages of tumor evolution (26–28). From this perspective, the stratification of patients according to their genetic profile constitutes a good example of personalized medicine. Such an approach is already being used successfully in breast cancer and is being extensively studied in other types of cancer, including head and neck cancer.

The results of the studies addressing the question of how the tumor gene expression profiles vary between the patients responsive and unresponsive to induction chemotherapy are collected in **Table 1**.

Zhong et al. (13) analyzed gene signatures in the pretreatment HNSCC tumor samples of 29 patients with advanced (TNM III or IV) hypopharyngeal cancer. The expression levels of 10 genes (GATS, PRIC285, ARID3B, ASNS, CXCR1, FBN2, INMT, MYOM3, SLC27A5, and STC2) out of 722 genes were identified as important for training the support vector machine model. Six genes (GATS, ARID3B, ASNS, FBN2, SLC27A5, and STC2) were overexpressed in the patients sensitive (defined as tumor volume decrease of approximately 70% after chemotherapy) to iCHT, while four genes (PRIC285, CXCR1, INMT, and MYOM3) were overexpressed in the non-sensitive (defined as the tumor volume decrease by less than approximately 25%) patients and the differences were statistically significant. The data from 21 patients were used as the training dataset, while the remaining 8 patients comprised the validation dataset. The model achieved 75.0% sensitivity and 100% specificity for the chemotherapy response prediction. The overexpression of two genes (CXCR1 and ARID3B) was confirmed by immunohistochemistry.

Hasegava et al. (14) examined the multigene mRNA expression in the pretreatment biopsy specimens from 64 TNM stage II, III, and IV oropharynx and hypopharynx cancer patients. All patients received induction chemotherapy with 5−fluorouracil and cisplatin. The log−transformed expression levels of 22 genes were compared in the complete (32.8% of patients) and non-complete responders using Student’s t-test. High expression levels of ERCC1, XPA, p53, Bcl-2, VEGF, MDR1, and DPD were significantly associated with the increased sensitivity to chemotherapy. Subsequently, the significant gene expressions were subjected to multivariate logistic regression, where the overexpression of ERCC1 (and T stage) was identified as an independent predictor of a favorable response to iCHT.

Yang et al. (15) aimed at the prediction of the chemo-sensitive biomarkers and found a correlation between the gene expression and the clinical staging and pathological grading. They used a real-time quantitative fluorescence PCR (RT-qPCR) to analyze the mRNA expressions of MAPK10, c-Jun, and Itga6 genes in the pretreatment samples of 57 laryngeal squamous cell carcinoma patients. The study group showed the following TNM tumor staging: II (30 patients), III (16 patients), 8 (IVa), and 3 (IVb). All patients received two cycles of TPF induction chemotherapy. Based on the response to iCHT, the patients were divided into the sensitive (a complete or partial response) or resistant (a stable or progressive disease) groups. Itga6 was overexpressed in the resistant group while MAPK10 and c-Jun were overexpressed in the sensitive group. Furthermore, the patients with lymph node metastases (N >0) and high TNM stage (III and IV) showed a lower expression of MAPK10 and an overexpression of Itga6.

As shown by Noman et al. (16) for an early diagnosis and prognosis of HNSCC, it is necessary to validate and explore the prognostic values of the key genes of the cancer. They used The Cancer Genome Atlas (TCGA) database analysis and performed the experimental validation of the widespread expression of Sonic hedgehog (Shh) and Nrf2 genes in HNSCC patients treated with cisplatin. Their goal was to determine whether this gene pair shows combined clinical significance in HNSCC and could be used to predict the response to cisplatin-based chemotherapy. The gene expressions were analyzed in the resected tumor samples and, based on the expression scores, the patients were split into two groups: the high and low expressions for Shh and Nrf2, respectively. From 183 patients in the study, 53 received iCHT (based on cisplatin and 5-fluorouracil); 25 of them showed a complete response, whereas the remaining 28 patients were resistant (the authors provided no details regarding the scoring of the response to iCHT). In the non-responders, a simultaneous upregulation of both genes was observed.

#### 3.1.2 Proteins

Protein expression levels, driven by various genes, bridge the gap between genotype and phenotype. Posttranslational modifications of proteins may result in the development of a disease. However, while a particular disease may be of genetic origin, protein expression reflects the functional consequences of the disease itself as well as the response of the immune system. Proteomic analysis plays an important role in cancer research, judging by the large group of publications analyzing protein expression as a predictor of treatment success. The summary of these protein-based studies on predicting the iCHT response is shown in **Table 2**.

Wang et al. (29) analyzed the pretreatment tumor tissue samples obtained from 102 hypopharyngeal SCC patients. The patients received TPF iCHT (the number of cycles is not given) and the treatment evaluation was assessed based on the contrast-enhanced CT. The patients who achieved CR or PR were classified as responders (n = 75), and those with SD and PD were the non-responders (n = 27). The expression of transcobalamin 1 (TCN1), a vitamin B12-binding protein, was analyzed and correlated with the iCHT response. The negative and weak immunohistochemical staining categories were grouped together and considered to have low TCN1 expression, whereas the moderate and strong staining categories were considered to have high expression. An overexpressed TCN1 was found in 16 non-responders and 24 responders, while in the group with a low TCN1 expression, there were 11 non-responders and 51 responders, and the difference was statistically significant. The authors confirmed the above findings by qPCR analysis of the isolated mRNA from 10 iCHT responders and 23 non-responders.

Lee et al. (30) investigated the prognostic value of p16 (cyclin-dependent kinase inhibitor 2A, postulated as a surrogate marker for HPV) and the tumor protein P53 expression with respect to the response to iCHT for advanced hypopharyngeal SCC. Forty-five patients with TNM III and IV were treated with three (5 patients received two cycles) cycles of TPF (n = 35) or PF (n = 10) iCHT. Seventeen patients achieved CR and 28 patients had PR. The pretreatment tumor tissue samples were analyzed immunohistochemically. The expressions of p16 and p53 were categorized as high or low (considering the staining intensities and the percentages of the staining areas for p16 and p53, respectively). Eleven patients were p16-positive and 30 patients had high p53 expression. The expressions of p16 and p53 were not correlated with each other. Furthermore, both expressions showed no differences in the iCHT response groups (CR vs. non-CR; PR and CR vs. other).

The immunohistochemical staining results for p16 and p53 proteins were also evaluated by Karpathiou et al. (31). Eighty-one LA HNSCC patients (TNM III and IV) were involved in their study. Sixteen patients had metastatic disease at the time of diagnosis. The patients were treated with up to two cycles of TPF iCHT and the tumor shrinkage was evaluated by CT and MRI as the decrease in the sum of the product of the largest perpendicular diameters of the measurable lesions at the primary tumor site. A good response to the treatment was defined as a decrease of at least 80% of the initial tumor size. According to such criteria, 50 patients were good responders to iCHT. The analyzed samples were binary classified for p16 (positive or negative), while three patterns of expression (overexpression, negative, and normal) were used for p53. The expression of both p16 and p53 showed no correlation with the response to iCHT.

Similarly, Ladányi et al. (32) observed no correlation between the expression of p16 and the response to iCHT evaluated by CT or MRI. They analyzed the pretreatment biopsy samples from 47 patients with locally advanced oropharynx, hypopharynx, larynx, or oral cavity cancer. The patients received two cycles of TPF plus cetuximab induction. There were 33 responders (PR) and 14 non-responders (SD + PD). Although 12 patients were p16 positive and 35 were p16 negative, no information about the distribution of the p16 status between the responders and the non-responders is given. The p16 status was significantly correlated exclusively with the tumor site (all oral cavity and larynx cancers were p16-negative).

The expression of p53 as well as that of pituitary homeobox 1 (PITX1) protein with regard to the iCHT response was analyzed by Takenobu et al. (33). Their study group consisted of 21 laryngeal, 16 hypopharyngeal, 5 oropharyngeal, and 5 oral SCC. Forty-one patients had stage III or IV of the disease, and all patients received one cycle of TPF iCHT. Of the 47 patients, 6 had CR, 21 had PR, and 19 showed SD. PD was observed in 1 patient. Immunohistochemically analyzed expressions of PITX1 and p53 were compared between the three groups of iCHT response: CR, PR, and SD + PD. The p53 expression showed no difference, while the CR patients had a significantly higher PITX1 expression than those with SD and PD. There were no differences in PITX1 expression between CR and PR as well as between PR and SD + PD.

Zhang et al. (34) evaluated the association between Notch1 (single-pass transmembrane protein encoded by the NOTCH gene) expression and clinical response to iCHT. They enrolled 72 patients with stage II (poorly differentiated tumors) or stage III/IV HNSCC. The patients received two cycles of PF (n = 34) or TPF (n = 38) iCHT and were categorized as responders when they achieved CR or PR, whereas those showing SD or PD were denoted as non-responders. There were 14 responders (3 CR and 11 PR) and 20 non-responders (14 SD and 6 PD) in the PF arm, while among the patients treated with TPF, 26 were responders (5 CR and 21 PR) and 12 were non-responders (8 SD and 4 PD); the difference in the response rate between PF and TPF was statistically significant. Notch1 expression in the pretreatment biopsies was quantified and categorized as either positive (strong or moderate staining) or negative (weak or no staining). The responders (in both PF and TPF groups) showed significantly lower Notch1 expression than the non-responders. Furthermore, Notch1 expression was significantly positively correlated with T and N stages.

Altuntaş et al. (35) assessed the usefulness of tumor expression of lysyl oxidase-like 4 (LOXL4) as a prognostic marker in advanced stage (T3 and T4) laryngeal cancer. Twenty-five of the 72 patients under study were treated with iCHT; they received three cycles of TPF induction. Twenty-one patients were responders (≥50% reduction in tumor diameter), and 4 patients had no response (salvage surgery after iCHT). The pretreatment laryngoscopic biopsy specimens underwent immunohistochemical analysis. LOXL4 expression was categorized as positive or negative (considering staining intensity and extent). Positive LOXL4 immunostaining was observed in 10 responders and 2 non-responders, while 11 responders and 2 non-responders were LOXL4 negative. There was no correlation between the LOXL4 expression and the response to iCHT, although a significant positive correlation with the T and TNM stages was observed.

Yang et al. (15), in addition to the mRNA expressions, used immunohistochemistry to detect the protein expression of MAPK10, c-Jun, and Itga6 genes. The immunohistochemically obtained results for MAPK10 and c-Jun were in agreement with the mRNA expression, whereas Itga6 was overexpressed in the responders and this result was inconsistent with the findings by microarray analysis. The authors suppose that this may be attributed to the post-transcriptional modification of mRNA.

#### 3.1.3 Diagnostic Imaging

Several non-invasive imaging techniques are used in cancer diagnostics. They can be divided into two broad categories: the methods that define anatomical details—Computed Tomography (CT) and Magnetic Resonance Imaging (MRI); and those that produce functional or molecular images—Single Photon Emission Computed Tomography (SPECT) and Positron Emission Tomography (PET). As CT is recognized as the “gold standard” for assessing morphological changes in the tissues due to cancer, MRI has been established as a radiation-free alternative to CT. However, it not only offers a superior contrast resolution between different types of soft tissues but also allows physiological (dynamic contrast enhanced MRI), metabolic (MR spectroscopy), and molecular (diffusion weighted imaging) phenomena to be observed. The other two imaging modalities, SPECT and PET, can detect and track the functional processes and metabolic changes due to carcinogenesis. In contemporary practice, contrast‐enhanced CT and MRI constitute the mainstay of imaging for treatment response assessment. However, there are various approaches for measuring the response rate, such as the World Health Organization (WHO) criteria (1979) (36), the European Organization for Research and Treatment of Cancer (EORTC) criteria for PET (1999) (37), the Response Evaluation Criteria in Solid Tumors (RECIST) (2000) (38), the National Cancer Institute guidelines (2006) (39), the RECIST 1.1 (2009) (12), and the PET Response Criteria in Solid Tumors (PERCIST) (2009) (40). These various classifications provide convenient factors useful in clinical practice.

Nowadays, diagnostic imaging is used in oncology to assess the disease spread, stage of advancement, tumor metabolism, radiotherapy planning, and for monitoring the treatment response, including that of iCHT. From a historical perspective, treatment efficacy rates for solid tumors are based on tumor size. However, functional imaging—e.g., FDG‐PET—potentially provides an earlier indication of the response to treatment than conventional imaging techniques. The applicability of the mentioned diagnostic imaging techniques for predicting iCHT response is shown in the papers collected in **Table 3**.

Gavid et al. (41) correlated the reduction in maximum standard uptake value (SUVmax) and in hypermetabolic tumor volume after the first iCHT cycle (compared to the pretreatment values) with the clinical response at the end of iCHT. Twenty-one T3 and T4 HNSCC patients with significant lymph node involvement (20 patients presented with N2 or N3 stage) were treated with two or three cycles of TPF chemotherapy (2 patients received only one cycle). Thirteen patients responded favorably with iCHT, showing a ≥70% reduction in tumor volume on the control endoscopy. The responders showed a significantly greater SUVmax reduction between the consecutive PET examinations, while the initial (before iCHT) SUVmax was similar in both groups. The difference in the reduction of the hypermetabolic tumor volumes was, however, statistically insignificant.

The lack of differences in the pretreatment values of the SUVmax and the hypermetabolic tumor volumes between the responders and the non-responders to iCHT was confirmed by Šedienė et al. (42). They studied a group of 35 HNSCC patients (mostly with T stage 3 and 4 and N+) treated with the TPF regime. Twenty-two patients had a complete or partial response (responders), whereas the remaining 13 patients demonstrated stable or progressive disease (non-responders). Because the PET/CT examinations were acquired before and after the completion of iCHT, the reduction in the SUVmax values and the metabolic tumor volumes could not be used as predictors of the response to iCHT.

Semrau et al. (43) and Wichman et al. (44) analyzed the PET/CT parameters acquired at baseline and after the first cycle of iCHT in terms of long-term criteria, i.e., tumor-free, laryngectomy-free, and overall survival. Sixty-two and 47 LA-HNSCC patients were involved in these studies, respectively. Both groups adopted an identical criterion for the distinction of the responders and non-responders, i.e., endoscopic tumor surface shrinkage of ≥30%. Based on the results presented, neither the baseline SUVmax nor the metabolic tumor volume was significantly different between the responders and non-responders after the first iCHT cycle.

MRI is another imaging modality offering an insight into the tumor morphology and biology (such as its metabolic activity and cellularity) *via* the specialized MR techniques, like T1- and T2-weighted imaging, *in vivo* proton magnetic resonance spectroscopy (*in vivo* 1H MRS), diffusion-weighted imaging (DW-MRI) or dynamic contrast-enhanced MRI (DCE-MRI). The latter one is based on a serial acquisition of images before, during, and after the introduction of a paramagnetic contrast agent to analyze the temporal enhancement pattern of a tissue. The degree of this enhancement depends, inter alia, on the regional blood flow as well as on the size, number, and permeability of the vessels. Because the changes in tumor metabolism appear early during therapy and precede the reduction in tumor size (49), MRI is useful for monitoring the response to iCHT.

Bernstein et al. (45) conducted a prospective open study to test the relationship between the response to iCHT and a baseline (three weeks before iCHT) tumor and lymph nodes: plasma flow (F_p_), endothelial permeability-surface area product, plasma volume, the volume of extravascular extracellular space (EES), and volume transfer constant. Thirty-seven stage IV HNSCC patients underwent the DCE-MRI examinations—they were defined as the responders or the non-responders according to the change in the aggregate RECIST dimension of the tumors and the lymph nodes. The patients were scheduled to receive three cycles of TPF chemotherapy. A partial response to iCHT was observed in 25 patients, whereas 12 patients presented with stable disease. No cases with complete responses or cases with progressive disease were observed. The baseline median F_p_ was significantly higher in the responders than in the patients with stable disease. However, while the higher tumor F_p_ values predicted a response, the lower values did not distinguish the responders from the non-responders. Approximately 25% of the responders presented the F_p_ values as being lower or comparable to the non-responders, which is reflected in the AUC = 0.73. Furthermore, a significant, yet weak, correlation was observed between the baseline F_p_ and the decrease in the sum of the linear dimensions of the primary tumor.

Ryoo et al. (46) applied DW-MRI in their studies of the response to iCHT. DW-MRI visualizes the internal physiology *via* the diffusion of water molecules; the image contrast reflects the difference in the rate of diffusion between the tissues. The study group included 25 HNSCC patients, 15 with T4 stage. All patients underwent two or three cycles of iCHT (TPF, or docetaxel and cisplatin with or without cetuximab). The complete (n = 2) and partial (n = 11) responders composed the responder group, while the non-responding group consisted of the patients with stable (n = 7) and progressive (n = 5) diseases. The response was evaluated approximately two weeks after the second iCHT cycle. The DW-MRI sequences were acquired at both standard (b = 0 and 1,000 s/mm^2^) and high (b = 0 and 2,000 s/mm^2^) b-values. The authors analyzed the tumor volumes (the volumetric calculations were performed based on the MRI images) as well as the following parameters derived from the obvious diffusion coefficient (ADC) histograms: mean, kurtosis, and skewness, as well as the tumor volumes, in terms of the favorable response to iCHT. The mean ADC values from the high b-value sequences (ADC_2,000_) were significantly lower in the responders than in the non-responders, and in the ROC curve analysis, the AUC value of ADC_2,000_ was 0.769 for predicting a good response to iCHT. The mean tumor volume (cm^3^) was significantly higher in the non-responders than in the responders. However, the ADC_2,000_ was the only significant predictor indicated by multiple logistic regression analysis. The remaining ADC parameters and a T stage, were not statistically significant.

Diagnostic imaging techniques join the “omic” cluster *via* radiomics. Radiomics assumes that biomedical images contain information about disease-specific processes that are imperceptible to the human eye and therefore may escape diagnosis (50, 51). The visual analysis of the tumor medical images is based on some qualitative descriptors (such as tumor tissue heterogeneity) or simple quantitative features (such as maximum diameter or volume)—such features can be extracted from the imaging data automatically with various computational methods. It does not mean the automation of the diagnostic processes, but rather provides clinicians with additional data through advanced mathematical analysis, by identifying the patterns characteristic of the tumor phenotype during and after treatment, and by identifying key predictive information (51).

Zhang et al. (47) used a CT-based radiomics model to predict a lymph node response to iCHT. The study group comprised 27 stage IVa or IVb HPV+ HNSCC patients. The patients were treated with two cycles of iCHT using cisplatin, paclitaxel, and escalating doses of cetuximab and everolimus. The response to iCHT of 41 enlarged (defined as a short axis diameter >15 mm) lymph nodes was measured as a percent change between the pretreated and posttreatment volumes. The median reduction in lymph node volume of 66% was used as the cutoff between a good/poor response. Ninety-three radiomic features were extracted from the regions of interest (ROI) on the pretreatment CT axial slices with the largest lymph node cross-sectional area. Two first-order features (minimum skewness) and one gray-level run length matrix feature (Low Gray level Run Emphasis, LGRE) were selected for further analysis after a three-step feature selection procedure. The logistic regression model showed, as the authors claim, that the minimum pixel intensity and LGRE were positively associated with a good lymph node response while the skewness was associated with a poorer lymph node response. However, these results were not statistically significant. The usefulness of the radiomics model (alone and combined with the clinical model involving age, sex, as well as T and N stages) was validated using a ROC curve analysis on the training (n = 30) and test (n = 11) sets. The combined (radiomics-clinical) modeling performed best, resulting in an AUC of 0.85 and 0.67 for the training and test sets, respectively. In the test set, the model was 100% sensitive and 50% specific.

The usefulness of other imaging modalities, such as the score of liver ultrasonography, in predicting the response to iCHT was evaluated by Wang et al. (48). Forty-seven (TNM stage III to IVB) LA-HNSCC patients underwent the liver ultrasonography examination within four weeks before the iCHT. The severity of hepatic parenchymal damage was assessed based on a scoring system including liver surface, parenchyma, vascular structure, and splenic size. A summed score ranged from 4 (normal liver) to 11 (advanced cirrhosis), and a score of 7 was chosen as the cutoff. All patients were treated with at least one cycle of TPF based iCHT (34 patients received at least three cycles of iCHT). CR and PR were observed in 3 and 22 patients, respectively, while 5 and 8 patients showed SD and PD, respectively. Nine patients died before our evaluation. The CR rate and the overall response rate (CR + PR) showed no significant correlation with a liver ultrasonography score.

#### 3.1.4 Clinicopathological Parameters

Many clinical, histologic, social, and demographic parameters are routinely (and/or as a part of a research project) recorded at diagnosis, treatment planning, and at the start of treatment as the baseline data for monitoring the condition of the patient and treatment response. All these parameters can be analyzed for their utility as prognostic markers. In this review, a list of the clinicopathological parameters effective in predicting iCHT responses is gathered in **Table 4**.

Besides the expression of p16 and p53 proteins, Karpathiou et al. (31) assessed the usefulness of several clinical and histological parameters in the prediction of the iCHT response. The characteristics of this study group and the evaluation of the iCHT response can be found in the previous section. The following parameters were analyzed: localization, stromal reaction, and histologic type of tumor; lymphocytic host response (LHR); neutrophil; and lymphocyte counts as well as neutrophil to lymphocyte (NLR) and platelet to lymphocyte (PLR) ratios; worst pattern of invasion (WPOI); and Brandwein–Gensler (B–G) risk. The good responders (n = 50 out of 81) were hypopharyngeal cancers (p = 0.01) with a dense LHR (p = 0.009), a lower (<150) PLR ratio (p = 0.03) and with a low/intermediate B–G risk score before the treatment (p = 0.002). The B–G, PLR, and LHR were not correlated with tumor localization.

Sánchez-Canteli et al. (52) analyzed the significance of NLR in peripheral blood and the immune infiltrate profiles (i.e., number of CD8+ and FOXP3+ tumor infiltrating lymphocytes (TIL) and programmed cell death ligand 1 (PD-L1) expressions) in the pretreatment biopsies to establish their potential relationship with the response to iCHT. 64 patients (58 men and 6 women) with stage III or IV larynx and hypopharynx SCC were included. The patients received a single cycle of iCHT with cisplatin and 5-Fluorouracil. A tumor response was defined by a decrease of at least 50% in the largest tumor dimension. Immunohistochemically assessed tumor PD-L1 expression was scored as: negative, low, intermediate or high, while CD8+ and FOXP3+ staining were automatically quantified and the mean values were statistically analyzed. From 58 patients with a partial response, 26 patients (41%) showed a response to iCHT greater than 50% in the larger dimension of the primary tumor—they were considered responders; a progressive and stable disease was observed in 1 and 5 patients, respectively. The rest, 32 patients, were considered partial responders (with a decrease in tumor size below 50%); none showed a complete response. None of the pretreatment hematological parameters (hemoglobin, leucocytes, neutrophils, and lymphocytes) was found to differ between the responders and the non-responders. However, a higher number of neutrophils was observed in the patients responding to iCHT, and a lower number of lymphocytes, and consequently, a higher NLR (calculated by dividing the absolute number of neutrophils by the number of lymphocytes), with a difference reaching a borderline statistical significance (p = 0.058). The PD-L1 tumor proportion score was not correlated with response to iCHT, while the positive combined proportion score (PD-L1 CPS) was significantly associated with a worse response to iCHT. The mean density values of TIL between the responders and the non-responders showed no differences. However, the mean ratio of CD8+ to FOXP3+ was significantly higher in the responders. Furthermore, CD8+ and FOXP3+ TIL showed a significant, yet weak (R ≈ 0.3), correlation with the PD-L1 combined proportion score. In a combined analysis, the patients with a negative PD-L1 CPS and a high CD8+ (n = 5) as well as a negative PD-L1 CPS and a high NLR (n = 9) exhibited the highest response rate (4 of 5 and 7 of 9, respectively, were the responders), whereas among the patients with a positive PD-L1 CPS and a low CD8+ (n = 20) as well as a positive PD-L1 CPS and a low NLR (n = 28), only 4 (20%) and 7 (25%) of the patients were the responders. The results obtained by a combination of PD-L1 CPS and NLR were statistically significant, while multivariate logistic regression identified the high NLR values as the only parameter independently associated with the response to iCHT.

The relationship between the inflammatory markers and chemo-sensitivity to iCHT was also investigated by Sun et al. (53). They analyzed the pretreatment counts of neutrophils (NC), platelets (PC), lymphocytes (LC), and monocytes (MC). The study group consisted of 72 hypopharyngeal SCC patients. Twenty-one patients had TNM stages I–III and 51 had TNM stage IV. Five patients achieved CR, 47 achieved PR, 19 had SD, and 1 had PD. 52 were assigned as the responders (CR + PR), and 20 as the non-responders (SD + PD). A significantly lower LC and higher values of NLR and PLR were reported in the responders compared to the non-responders. An ROC curve analysis showed that these three parameters were the significant predictors of a good response to iCHT also among the stage I–III and stage IV cohorts analyzed separately.

A group from the same hospital evaluated the chemo-sensitivity to iCHT in hypopharyngeal SCC by analyzing the values of different immune cells (54). Forty patients (13 patients with stage I–III disease and 27 patients with TNM stage IV) were treated with three cycles of TPF iCHT. Twenty-six patients were responders (CR + PR), and 14 were non-responders. Four immune cell indicators (CD4+ T cells, CD8+ T cells, Tregs, and NK cells) were defined using multicolor flow cytometry on the pretreated peripheral blood mononuclear cells. The CD4+ T-cell and CD8+ T-cell frequencies were significantly higher in the responders, whereas the Treg frequencies were significantly lower. The CD4+ T-cell frequencies were also significantly lower in the patients with stage IV disease compared to the TNM stage I–III group.

Besides the p16 status, Ladányi et al. (32) evaluated the infiltration levels of various immune cell types in association with the response to iCHT. The characteristics of the study group are available in the *Proteins* section and **Tables 2**, **4**. Between 35 and 40 pretreatment biopsy samples with a sufficient amount of tumor tissue were available. The immune cell infiltration was assessed using immunohistochemistry. The tumor-associated immune cell types (CD8+ and CD45RO+ T cells, CD20+ B cells, lymphocytes expressing the activation markers CD134, CD137, or PD-1, FOXP3+ regulatory T cells, NKp46+ NK cells, CD68+ macrophages, cells expressing CD16, myeloperoxidase+ neutrophil granulocytes (MPO)), DC-LAMP+ mature dendritic cells were identified in the analyzed samples. A strong and diffuse nuclear and cytoplasmic staining in ≥70% of tumor cells was scored as a positive result. Additionally, the peripheral blood neutrophil and lymphocyte counts were also analyzed. The responders showed a significantly higher incidence of the positive DC-LAMP and PD-1 cells and a higher pretreatment NC compared to the non-responders.

The prognostic role of circulating tumor cells (CTCs) during iCHT was assessed by Inhestern et al. (55). Forty patients with oral or oropharynx SCC were included. The tumor staging was T2 (n = 15), T3 (n = 15), and T4 (n = 10). Thirty-five patients had the N3 stage. The patients were treated with three (n = 24), two (n = 1), and one (n = 15) cycles of TPF iCHT. Eight and 24 patients had complete or partial responses, respectively, whereas 6 patients showed a stable disease and 2 progressed. Although the primary aim of this study was to correlate the CTCs with recurrence-free and overall survival, in the supplementary material the authors showed that the baseline (before the 1st iCHT cycle) CTC levels were noy statistically different (p = 1) between the CR and not-CR patients.

Zhao et al. (56) investigated the role of BMI in the prognosis of TPF iCHT in 109 patients with locally advanced (TNM stage III and IV) oral SCC. The patients were stratified into four BMI groups (the values for Asian population): underweight (BMI <18.5 kg/m^2^); normoweight (18.5≤ BMI <23.5 kg/m^2^); overweight (23.5≤ BMI <27.5 kg/m^2^); and obese (BMI ≥27.5 kg/m^2^), the measurements were made when the patient initially arrived at the hospital. There were no significant differences in the clinical responses to iCHT between the four BMI groups. However, the study was focused on overall survival, and thus, no detailed information regarding the iCHT response was given.

It has been widely postulated that disease advancement is a negative prognostic factor for iCHT response. However, the results in the reviewed articles are inconsistent on this matter. TNM stage (III, Iva, IVb, and IVc) was found to be of no statistical significance (13), while the higher T stage was significantly correlated with a poorer response in one article (14). However, two studies showed no correlation between T and the response to the treatment (46, 57). In the case of the role of the nodal stage, the results were contradictory: no correlation was found in (14) while in (57) identified the higher N stage (>1) as an important prognostic factor.

#### 3.1.5 Demographic and Social Factors

In some studies, social, demographic, and environmental factors, such as age, gender, smoking, and diet (mainly alcohol consumption), were taken into account in the analyses. However, none of these factors were revealed to be predictive of iCHT efficacy (13, 35, 46, 53). Only betel nut chewing history among the smoking HNSCC patients was reported as a significant predictor for a poorer iCHT response (57).

### 3.2 Identification of Prognostic Factors for Toxicity of Induction Chemotherapy

The main factor limiting both the dose and the number of cycles in iCHT is treatment-induced toxicity. TPF schedule demonstrates a better response rate than PF, but due to its less favorable toxicity profile, a PF arm is preferred in the patients with poorer performance status or burdened with a severe comorbidity and in age >70 years (58, 59). Standard iCHT protocols involve the administration of 3 to 4 cycles (2, 59), however, it has been recently shown that two cycles of iCHT might be sufficient in case of nasopharyngeal cancer and additional more cycles did not lead to survival benefit (60). The adverse iCHT effects may appear even after the first cycle, having a significant negative impact on the patient’s ability to undergo therapy (61).

In this review, the primary focus is to collect the predictive markers of toxicity and pathological response to iCHT. Accurate judgment of the onset of the iCHT treatment-related acute toxicity seems to be of importance, especially when considering the aggressiveness of iCHT as well as its social and economic costs. The Common Terminology Criteria for Adverse Events (CTCAE) (62), a standardized scoring system for classification toxicity in cancer therapy, was adopted for assessment of the analyzed adverse events due to iCHT in the papers taken into account in this review. Since the same types of toxicity were analyzed in the context of different predictors in the reviewed studies, the results are collected in a single table (**Table 5**), which makes it much easier to get a broader view of the problem.

**Table 5:** Summary of predictive factor for iCHT toxicity.

Predictive factor		Toxicity											
		Anemia	Anorexia	Febrile neutropenia	Gastrointestinal toxicity	Hematological toxicity	Hyponatremia	Infection	Nephrotoxicity	Neutropenia	Thrombocytopenia	Whole side effects	Treatment completion
rs8187710	SNP of ABCC2 gene				↑ (63)								
rs1801131	SNP of MTHFR gene				↑ (63)								
rs3788007	SNP of ABCG1 gene				⇵ (63)								
rs4148943	SNP of CHST3 gene				⇵ (63)								
rs2301159	SNP of SLC10A2 gene					↑ (63)							
rs2470890	SNP of CYP1A2 gene					↑ (63)							
SLU	Score of liver ultrasonography			↑ (31)						↑ (48)			
SI	Spleen index									ns (48)			
Older age		↑ (64)		ns (64, 65)				ns (64)	ns (64)	ns (48, 64)	↑ (64)		ns (66)
Sex		ns (64)		ns (64, 65)				ns (64)	ns (64)	ns (64)	ns (64)		ns (66)
BMI	Body mass index			ns (65)						ns (48)			↑ (66)
Localization	Primary tumor localization	ns (64)		ns (64, 65)				ns (64)	ns (64)	ns (48, 64)	ns (64)		* (66)
T	Primary tumor stage	ns (64)		ns (64, 65)				ns (64)	ns (64)	ns (64)	ns (64)		ns (66)
N	Nodal stage	ns (64)		ns (64, 65)				ns (64)	ns (64)	ns (64)	ns (64)		ns (66)
T + N		ns (64)		ns (64)				ns (64)	ns (64)	ns (64)	↑ (64)		
TNM	Clinical disease stage			ns (65)						ns (48)			
ALT	Alanine aminotransferase			ns (65)									
ALB	Albumin		ns (67)	ns (65, 67)			ns (67)					ns (67)	
AST	Aspartate transaminase			ns (65)									
BUN	Blood urea nitrogen			ns (65)									
CCr	Creatinine clearance			ns (65)									
CAR	C-reactive protein to albumin ratio		↑ (67)	↑ (67)			↑ (67)					↑ (67)	
CRP	C-reactive protein		ns (67)	ns (65, 67)			ns (67)					ns (67)	
LC	Lymphocyte count			ns (65)						ns (48)			
LDH	Lactate dehydrogenase			ns (65)									
LMR	Lymphocyte to monocyte ratio			ns (65)									
mGPS	Modified Glasgow prognostic score		↑ (67)	↑ (67)			↑ (67)					↑ (67)	
MC	Monocyte count		ns (67)	⇵ (65)ns (67)			ns (67)					ns (67)	
NC	Neutrophil count		ns (67)	⇵ (65) ns (67)			ns (67)			ns (48)		ns (67)	
PC	Platelet count		ns (67)	ns (65, 67)			ns (67)			ns (48)		ns (67)	
NLR	Neutrophil to lymphocyte ratio			ns (65)						ns (48)			
PLR	Platelet to lymphocyte ratio			ns (65)									
WBC	White blood cells		ns (67)	⇵ (65) ns (67)			ns (67)			ns (48)		ns (67)	
Weight loss	Weight loss before iCHT	ns (64)		ns (64)				↑ (64)	ns (64)	ns (64)	ns (64)		
General condition		ns (64)		ns (64)				ns (64)	ns (64)	ns (64)	ns (64)		
Diabetes		ns (64)	ns (67)	ns (64, 65, 67)			ns (67)	ns (64)	ns (64)	ns (64)	ns (64)	ns (67)	
Tobacco/Alcohol		ns (64)		ns (64)				ns (64)	ns (64)	ns (64)	ns (64)		ns (66)
Hepatopathy		ns (64)		ns (64)				ns (64)	ns (64)	ns (64)	ns (64)		
Arterial hypertension		ns (64)		ns (64)				ns (64)	ns (64)	ns (64)	ns (64)		
Heart disease		ns (64)	ns (67)	ns (64, 67)			ns (67)	ns (64)	ns (64)	ns (64)	ns (64)	ns (67)	
COPD	Chronic obstructive pulmonary disease	ns (64)		ns (64)				ns (64)	ns (64)	ns (64)	ns (64)		
Feeding tube	Tube nutrition	ns (64)		ns (64, 65)				ns (64)	ns (64)	ns (64)	ns (64)		

ns, non-significant; ↑, higher value predicts increased toxicity; ⇵, higher value predicts lower toxicity; (), reference.

*Hypopharyngeal/laryngeal primary tumor site was a negative prognostic factor for treatment completion.

#### 3.2.1 Genes

De Marchi et al. (63) investigated 366 clinically relevant single-nucleotide polymorphisms (SNPs) on 47 metabolic or transporter genes to find an association between SNPs and toxicity to induction chemotherapy. The study group consisted of 59 LAHNSCC (TNM III and IV) patients treated with three cycles of iCHT with cisplatin and paclitaxel. The toxicities were classified and graded according to the CTCAE v3.0. The following toxicities were investigated: peripheral neuropathy, infectious complications, hematologic toxicity (febrile or afebrile neutropenia or anemia or lymphopenia or thrombocytopenia), and gastrointestinal toxicity (nausea or vomiting or diarrhea or constipation). The incidences of toxicities in ≥grade 2 were correlated with the selected SNPs. Multivariate logistic regression analysis revealed that rs8187710 (gene ABCC2) and rs1801131 (gene MTHFR) were associated with the increased gastrointestinal toxicity, whereas rs3788007 (gene ABCG1) and rs4148943 (gene CHST3) were associated with the decreased risk. The increased risk of hematological toxicity was associated with rs2301159 (gene SLC10A2) and rs2470890 (gene CYP1A2). However, none of these SNPs were significant after adjusting for multiple comparisons. Infectious complications, nephrotoxicity, and neurological toxicity were observed in an insufficient number of patients (two, one, and none, respectively), so no associations with the SNPs were found.

#### 3.2.2 Diagnostic Imaging

Wang et al. (48) evaluated the usefulness of liver ultrasonography in the prediction of iCHT-related anemia, thrombocytopenia, mucositis, neutropenia, and febrile neutropenia. The study group consisted of 47 (TNM stage III to IVB) LA-HNSCC patients who, according to the treatment protocol, were planned to be treated with 3–4 cycles of TPF every 3 weeks. All of them obtained at least one cycle of TPF-based iCHT. The treatment-related adverse events were graded according to CTCAE v4.0. Liver ultrasonography was performed within four weeks before the iCHT and the severity of hepatic parenchymal damage was assessed on the basis of the scoring system, including liver surface, parenchyma, vascular structure, and splenic size. A summed score ranged from 4 (normal liver) to 11 (advanced cirrhosis), and a score of 7 was chosen as the cutoff. Univariate statistics showed that the ultrasonography score of ≥7 as well as the lower white blood cells (WBC) and platelet counts were significantly associated with severe and febrile neutropenia. However, multivariate logistic regression identified only the ultrasonography score of ≥7 as an independent factor that was significantly predictive of severe and febrile neutropenia.

#### 3.2.3 Clinicopathological Parameters

Shimanuki et al. (65) were looking for the predictors of febrile neutropenia development in 50 LA-HNSCC patients (45 patients had TNM stage IV). The patients received from 1 to 4 cycles of TPF chemotherapy as induction (n = 45) or as a first-line treatment for recurrent or metastatic tumors (n = 5). The following pretreatment hematological and biochemical laboratory parameters were analyzed: WBC, NC, LC, MC, NLR, PLR, lymphocyte to monocyte ratio (LMR), aspartate transaminase (AST), alanine aminotransferase (ALT), lactate dehydrogenase (LDH), blood urea nitrogen (BUN), creatinine clearance (CCr), and CRP. The CTCAE scale v4.0 was used to grade the adverse effects (except febrile neutropenia) in the first cycle of TPF chemotherapy, and the incidences of grade 3 or higher febrile neutropenia, neutropenia, anemia, thrombocytopenia, diarrhea, hyponatremia, hypokalemia, hyperkalemia, as well as increased ALT and creatinine levels were observed. However, only the febrile neutropenia, observed in 12 patients, was taken under further investigation. Univariate analyses showed that lower WBC, NC and MC were significantly associated with the incidence of febrile neutropenia, whereas MC was the only independent significant predictor identified by multivariate logistic regression. Furthermore, the authors showed that a prediction model comprising a combination of MC and NC (although NC alone was not correlated with febrile neutropenia) demonstrated superior diagnostic performance in the prediction of the development of febrile neutropenia compared to that of the model using MC alone.

Bernadach et al. (64) studied the TPF induction toxicity in 57 LA-HNSCC patients (39 patients with TNM stage IV, 18 with stage III, and 43 patients had N stage >0). During iCHT, the patients presented with grade 3 or higher anemia (scaled with CTCAE v4.0), neutropenia, febrile neutropenia, thrombocytopenia, infection, nausea, mucositis, diarrhea, and digestive hemorrhage. The following baseline clinical factors were significantly assessed with the increased (grade ≥3) treatment toxicities: age (age ≥57 years or older) with thrombocytopenia and anemia; higher T and N stage with thrombocytopenia; weight loss with infection.

Mikoshiba et al. (67) analyzed the treatment-related side effects after the first cycle of TPF-based iCHT. The study group consisted of 54 oropharyngeal and hypopharyngeal LA-HNSCC cancer patients. The incidences of severe (grade ≥3 according to CTCAE v4.0) leucopenia, neutropenia, febrile neutropenia, anorexia, diarrhea, nausea/vomiting, as well as the increases in creatinine, AST, ALT, hyponatremia, and lung infection were observed during the treatment. The usefulness of baseline CRP to albumin ratio (CAR), NLR, PLR, and modified Glasgow Prognostic Score (mGPS) as the predictors of severe iCHT toxicity was evaluated. The univariate and multivariate statistical analyses revealed that the high CAR and mGPS values were the independent prognostic factors of overall severe side effects (grade 4), febrile neutropenia, and hyponatremia.

An approach based on evaluating the potential factors predictive of the completion of TPF induction treatment (defined as ≥3 cycles administered) was chosen by Nakano et al. (66). Ninety-three LA-HNSCC patients with oropharynx, hypopharynx, larynx, oral cavity, paranasal sinus, nasopharynx, and an unknown primary site were enrolled in the study. The following clinical factors were correlated with TPF completion: sex, smoking history, age, BMI, T stage 4, N stage 3, and origin of the primary lesion. The patients who had their TPF schedule changed or terminated before the accomplishment of the three cycles were defined as having TPF failure. Seventy-three patients achieved therapy completion. Fisher’s exact test and logistic regression analysis revealed that BMI ≥22 was a positive prognostic factor and hypopharyngeal/laryngeal primary tumor site was a negative prognostic factor for TPF completion.

## 4 Discussion

Clinical management decisions for cancer patients are increasingly being guided by prognostic and predictive markers. A tumor biomarker is a tool that aids the clinician in answering clinically relevant questions regarding cancerous diseases. A more specific definition characterizes a tumor biomarker as a molecule (or a set of molecular compounds), a process, or a substance that is altered quantitatively or qualitatively in pre-cancerous or cancerous conditions (68). To be clinically useful, such biomarkers should be detected by one or more assays or tests should be accurate, reproducible, and reliable. Large (to assess rare events and subgroup effects) and generalizable studies must provide clinically more relevant choices. While the comparative effectiveness studies are based on observational research, randomized trials, and decision analysis (69) and, as such, can summarize and evaluate the evidence, the great majority of the studies are not randomized. Many HNSCC biomarkers have already been suggested to significantly impact diagnosis and prognosis. As revealed from this systematic inspection of the papers published between 2015 and 2021, there are more than one hundred independent parameters analyzed for their suitability as prognostic markers in HNSCC patients undergoing induction chemotherapy. The utility of many of these markers has already been shown in subsequent studies to be questionable or low. Some are promising but have not yet been introduced because they lack important features, such as high specificity and sensitivity, low cost, high positive predictive value, clinical relevance, and short turnaround time (70). The experimental limitations, like small sample sizes or unreliable data, the selective reporting and incomplete reporting literature on tumor markers, make it difficult to distinguish between the weak and strong biomarkers.

A good example of the former group is the tumor suppressor protein p53 (11, 14, 30, 31, 33). The conclusion about the unsuitability of p53 as a prognostic marker of iCHT efficacy appeared already in the mentioned review by Cosway et al. (11)—and six years later, in this review of the papers, it remains valid. The prognostic utility of the p53 gene in iCHT monitoring appeared only once (14); its significance was neither confirmed with multivariate logistic regression (14) nor by other studies (30, 31, 33). This summary of the “state of the art” in the field of iCHT response monitoring in head and neck cancer patients shows even more negative findings reported by multiple authors, e.g., p16 protein (30–32) or FDG PET maximum-standardized uptake value (42–44) as well as inconsistent findings, e.g., primary tumor or nodal stage (14, 46, 57).

Among the limitations of some of the reviewed studies is the rather small (<40) size of the study groups (13, 35, 41, 42, 45–47, 50, 51) or clear disproportion in the size of the compared groups (35, 55). An appropriate sample makes the research more efficient. The main problem with small patient populations is the interpretation of the results, in particular of the confidence intervals and p-values, which are then claimed to show “the trend” (71).

Another type of bias results from a practice designated as “incomplete study reporting.” Such reporting, which omits certain details, may lead to incorrect interpretation of the results, which, in turn, biases the analyses and would prevent other researchers from reproducing the study findings. Henry and Hayes (72) provide an example of how incomplete reporting of specific types of chemotherapy received by patients with breast cancer could lead to different conclusions about the direction of the association between HER2 status and chemotherapy efficacy. In some of the HNSCC studies (45, 46), such “restricted methodology” is also used.

Moreover, all the reviewed studies were retrospective and none of the reviewed studies (except (13, 47)) used an external test group to validate the predictive models. Most of the negative findings relate to the diagnostic imaging methods. Five of the reviewed studies analyzed the output data from FDG PET and/or CT and no significant correlations were found, except one study analyzing the reduction of the maximum standardized uptake value after the first iCHT cycle (thus with limited predictive value). However, it was conducted on an insignificant group of patients (41). However, the positive results were reported with DCE- (45) and DWI- (46) MRI, although the results of the latter study were also obtained in a small group of 25 patients. The high value of plasma flow in tumors as measured by DCE-MRI was observed in the responders, and according to the authors, such a finding suggests that the tumor perfusion could be of importance for the efficient delivery of low molecular weight chemotherapy agents (45). A similar conclusion has been drawn based on DWI-MRI, where the authors state that tumors with high intracellular water components, i.e., with increased cellularity, can show a better response to iCHT (46).

The reviewed protein-based studies were usually conducted on larger groups of patients, and six proteins were reported as important for the prediction of iCHT response. The low or negative expressions of TCN1 and Notch1 as well as the overexpressions of PITX1, MAPK10, c-Jun, and Itga6 were correlated with a better response to iCHT. Each of these proteins is involved in different metabolic and/or signaling pathways, and drawing direct biological conclusions is difficult. TCN1 is important in vitamin B12 homeostasis (29), Notch1 protein has diverse roles including differentiation and regulation of normal and cancer cells (34), PITX1 is essential in organ development processes (33), MAPK10 and c-Jun are involved in c-Jun N-terminal kinase 3 which is assumed to act as an inhibitor on tumor cell proliferation (15) and Itga6 is involved in cell division, migration, and invasion (15). The reviewed studies indicate that, despite being involved in such different processes, most of these proteins show significant correlations with disease advancement. A positive correlation with the severity of the disease was reported for the expressions of TCN1 (histological grade) (29), Notch1 (T and N stage) (34), while a negative correlation was observed for MAPK10, Itga6 (TNM stage, histological grade, and lymph node involvement), and c-Jun (TNM stage, histological grade) (15). PITX1 expression was significantly lower in tumors compared to healthy mucosa as well as it was lower in moderately and poorly differentiated tumors compared to those well differentiated, but the difference was not statistically significant (33). Furthermore, the levels of PITX1 expression were significantly different between the complete responders and those with a stable or progressing disease, while no difference was observed for a partial response (33).

Taking into account that disease advancement may have an impact on clinically observed regression after iCHT, the prognostic value of the presented markers has to be taken with caution.

The genetic studies revealed 22 candidates for prognostic markers. There are several cancer-related processes in which these genes are involved, and some of them (e.g., CXCR1) have multiple functions. ARID3B, CXCR1, GATS, Itga6, Shh, SLC27A5, STC2, and VEGF play an important role in cell proliferation and tumor progression or metastasis. The exact functions of these genes are not always obvious, e.g., contrary information about the ARID3B are reported (44). It has been postulated that different cellular localizations of ARID3B may result in different roles of this gene (73). In the study by Zhong et al. (13) ARID3B is overexpressed in the responders but the authors have not explained in detail the presented results. Another controversy is related to the STC2 gene, which is also upregulated in the responders (13), while other studies indicate that STC2 is a positive regulator of metastasis and poorer prognosis in HNSCC (74) and pancreatic cancer (75). The genes known as tumor suppressors or involved in tumor suppression mechanisms (FBN2, MAPK10, c-Jun, and p53), those involved in the nucleotide excision repair pathway (ERCC1, XPA), and the regulatory component of the apoptotic pathway (Bcl-2) were found to be overexpressed in the responders (13–15). The non-responders showed higher expressions of CXCR1 and PRIC285 (13), the genes that activate the inflammatory system and inhibit host immune responses. CXCR1 and its role in cancer is well documented in the literature (76). However the functions of PRIC285 are still not well explained. HNSCC patients with overexpression of DPD and MRP1 are at a lower risk of failure of chemotherapy, and it is not a surprise that these two genes are overexpressed in the responders (14). The responders also showed overexpression of ASNS (13). However, high expression of this gene is indispensable for oral SCC progression (77) and adaptation to nutrient deprivation and hypoxia (78), and may inhibit apoptosis. The other two genes, Nrf2 and Itga6, known to be promoting factors of chemoresistance, are overexpressed in the non-responders (15, 16). The non-responders also showed overexpression of INMT and MYOM3, but their function in HNSCC remains unknown.

Three out of four reviewed genetic studies conducted research on groups of >50 patients (14–16), and one study analyzed a smaller group of <30 patients (13). Nevertheless, the latter study used an external group (n = 8 patients) to validate their model and received approximately 75.0% sensitivity and 100% specificity for an iCHT response prediction.

The last group of the markers analyzed for their predictive usefulness were the clinicopathological parameters. Since such markers appear in many studies, it enables the identification of those that show similar trends and gives an opportunity to evaluate the consistency of the presented results. However, despite the relatively large number of statistically significant findings, the majority of the results were inconclusive. In HNSCC, the induction treatment is almost exclusively administered in advanced stages of the disease, thus the TNM staging was not significant (13). The T and N stages, when analyzed separately, were nonsignificant except in (14) and (57), where, respectively, a higher T and a higher N were observed in the non-responders. Among the standard laboratory blood parameters, platelet and monocyte counts were not significant, while contradictory results were presented for neutrophil and lymphocyte counts as well as the platelet (PLR) and neutrophil (NLR) to lymphocyte ratios (31, 32, 52, 53). Most of the analyzed immune cell types were also nonsignificant, except CD4 and CD8 lymphocytes and the CD8/FOXP3 ratio, which were higher in the responders (52, 54). However, CD4 was analyzed in only one study (54), while two other studies showed that CD8 and FOX3P alone were not significant (32, 52). The remaining parameters were analyzed by individual studies where the responders showed a higher incidence of positive DC-LAMP and PD-1 cells (32) and a higher lymphocytic host response (31) while the non-responders were the patients with a higher PD-L1 combined proportion score, an NLR to PD-L1 CPS ratio (52) and a Brandwein–Gensler risk score (31). One study reported that the hypopharynx is a favorable localization for iCHT response (31).

Six of the reviewed manuscripts aimed to identify the prognostic factors for iCHT toxicity. The following toxic side effects were analyzed: anemia, anorexia, neutropenia, febrile neutropenia, gastrointestinal and hematological toxicity, hyponatremia, infection, nephrotoxicity, and thrombocytopenia. Febrile neutropenia and neutropenia, including hematological toxicities as a whole, were the most frequent side effects and were analyzed in five of the reviewed manuscripts. Side effects as a whole as well as treatment completion were also under investigation. Only 24 out of over 160 analyzed relationships between particular toxicities and candidates for predictive markers were statistically significant (**Table 5**). Similarly to the treatment response, the clinicopathological factors were mostly nonsignificant or ambiguous (monocyte and neutrophil counts, white blood cells). Despite the insignificance of the CRP and albumin levels, an increased CRP to albumin ratio and a modified Glasgow prognostic score (a score based on levels of CRP and albumin) were positively correlated with the incidences of high grade anorexia, febrile neutropenia, hyponatremia, and the side effects as a whole (67). An older age was correlated with anorexia and thrombocytopenia, with a weight loss before iCHT with infection and a T plus N stage with thrombocytopenia. One of the studies identified single nucleotide polymorphisms of several genes as possible prognostic factors for hematological and gastrointestinal toxicities (63) and a score of liver ultrasonography was positively correlated with febrile neutropenia and neutropenia but not with the response to iCHT (**Table 3**) (48). Higher BMI was positively correlated with treatment completion, while hypopharyngeal and laryngeal primary tumor sites were negative prognostic factors for treatment completion (66). These findings are in contrast to the studies on response to iCHT where BMI was nonsignificant and hypopharyngeal tumor location was favorable for iCHT response (31). Although the study groups were larger, with around 50 patients, sometimes the high grade toxicities were relatively rare (<10), resulting in significant disproportions between the analyzed subgroups and, thus, statistical inference based on such data must be taken with caution.

## 5 Summary

The role of iCHT before radiotherapy or before chemo-radiotherapy is still debated, as the data on its efficacy are somehow confusing, but in laryngeal and hypopharyngeal cancer, it is one of the recommended organ preservation strategies. Response assessment after induction chemotherapy is currently probably most valuable if a choice must be made between an organ‐preservation approach (radiotherapy with or without chemotherapy) and surgery, particularly for hypopharyngeal and laryngeal cancer.

Compared to Cosway et al. (11), this review covers a broader spectrum of the parameters assessed for their predictive ability, such as those identified in imaging diagnostics, as well as new genetic and clinicopathological factors. This means constant progress in the search for new prognostic factors for the better prediction of the iCHT response. Nevertheless, the current stage of the research is similar to that outlined by Cosway et al. (11): despite the large number of proposed predictive markers, none is currently in clinical use or validated in a large cohort. Most of the positive results discussed here are from single-study analyses, and no comparative studies are available. Finally, there are no clear conclusions concerning the parameters analyzed by several groups. Therefore, further research is needed to identify the validated parameters that predict the response to induction chemotherapy and can be used to select the final therapy with less toxicity.

## Author Contributions

ŁB designed the study, conducted the literature search and extracted relevant publications, prepared and discussed the findings, and wrote the manuscript.

## Conflict of Interest

The authors declares that the research was conducted in the absence of any commercial or financial relationships that could be construed as a potential conflict of interest.

## Publisher’s Note

All claims expressed in this article are solely those of the authors and do not necessarily represent those of their affiliated organizations, or those of the publisher, the editors and the reviewers. Any product that may be evaluated in this article, or claim that may be made by its manufacturer, is not guaranteed or endorsed by the publisher.
